# Post-traumatic stress disorder in patients with unintentional acute poisoning and its influencing factors

**DOI:** 10.3389/fpubh.2026.1781276

**Published:** 2026-07-07

**Authors:** Jie Shi, Jianjian Liu, Tianzi Jian, Yingying Zheng, Xiangdong Jian, Baotian Kan

**Affiliations:** 1 School of Nursing and Rehabilitation, Cheeloo College of Medicine, Shandong University, Jinan, Shandong, China; 2 Department of Nursing, Qilu Hospital, Cheeloo College of Medicine, Shandong University, Jinan, Shandong, China; 3 Department of Poisoning and Occupational Diseases, Emergency Medicine, Qilu Hospital of Shandong University, Cheeloo College of Medicine, Shandong University, Jinan, Shandong, China; 4 Department of Occupational and Environmental Health, School of Public Health, Cheeloo College of Medicine, Shandong University, Jinan, Shandong, China; 5 Department of Hematology, Qilu Hospital, Cheeloo College of Medicine, Shandong University, Jinan, Shandong, China

**Keywords:** acute poisoning, influencing factors, post-traumatic stress disorder, self-efficacy, social support

## Abstract

**Introduction:**

The population of patients with unintentional acute poisoning is large, yet clinicians pay relatively little attention to the psychological state of this group. This study aimed to investigate the current status of post-traumatic stress disorder (PTSD) and its main influencing factors in patients with unintentional acute poisoning and provide data support and a theoretical basis for the prevention of PTSD and reduction of its incidence in this population.

**Methods:**

Convenience sampling was used to select 312 patients with unintentional acute poisoning who were admitted to a tertiary hospital in Shandong Province between January 1, 2022, to September 30, 2023. Data were collected using the general information questionnaire, Poison Severity Score, General Self-Efficacy Scale, Simple Coping Style Questionnaire, shortened Chinese version of the Family Resilience Assessment Scale, Perceived Social Support Scale, and PTSD Checklist. Statistical analyses included t-tests, one-way ANOVA, correlation analysis, and multiple linear regression.

**Results:**

The PTSD positivity rate among the patients with unintentional acute poisoning was 27.56%. Social support, general self-efficacy, and compensation insurance were protective factors against PTSD in these patients, whereas chronic medical history and poisoning severity were risk factors. Multiple regression analysis revealed that these factors collectively explained 88.5% of the variance in the PTSD total scores based on the results of multiple regression analysis.

**Discussion:**

The incidence of PTSD is high in patients with unintentional acute poisoning. Early clinical assessment is warranted, especially for those with severe poisoning and chronic disease. Active interventions to enhance self-efficacy and social support should be implemented.

## 1 Introduction

Acute poisoning is a common critical illness encountered in emergency departments. It is characterized by sudden onset, severe symptoms, complex conditions, and rapid progression. It can cause physical damage and psychological disorders such as post-traumatic stress disorder (PTSD) (1). Unintentional poisoning is one of the common causes of acute poisoning (2, 3). The Diagnostic and Statistical Manual of Mental Disorders, 5th Edition (DSM-5), published by the American Psychiatric Association in May 2013, identifies unintentional acute poisoning as a traumatic event that can increase the risk of PTSD (4). PTSD is a persistent psychiatric disorder that occurs after experiencing or witnessing extreme traumatic events, which is a delayed-onset and persistent mental disorder that follows severe traumatic events and can significantly disrupt physical and mental health and social functioning (5). Domestic and international studies have reported varying PTSD incidence for different traumatic events. However, systematic research on PTSD in patients with unintentional acute poisoning is limited (6, 7). The existing literature on poisoning and PTSD primarily focuses on intentional self-harm behaviors such as suicide attempts, with study populations mainly comprising patients with intentional poisoning, whereas systematic research on PTSD in patients with unintentional acute poisoning remains notably scarce. Unintentional acute poisoning often results from accidental events such as accidental pesticide ingestion, drug overdose, and chemical spills or exposure. Patients experience intense life-threatening experiences, which theoretically possess the traumatic foundation to induce PTSD. Moreover, since such patients are typically first admitted to emergency departments, physical symptoms remain the focus of clinical management, while the identification and follow-up of psychological trauma are highly prone to being overlooked. This study aimed to determine the prevalence of PTSD in these patients and analyze its influencing factors to provide evidence for proactive intervention.

## 2 Participants and methods

### 2.1 Participants

The patients with unintentional acute poisoning who were admitted to the emergency department of a tertiary hospital in Shandong Province from January 2022 to September 2023 were enrolled using convenience sampling. The inclusion criteria were clinically confirmed acute poisoning; age of ≥ 18 years; no history of alcohol or drug abuse; and ability to communicate independently and understand the questionnaire content. The exclusion criteria were intentional poisoning (patients with documented suicidal intention such as suicide note, confirmed by relatives or psychiatric evaluation or evidence of deliberate ingestion/exposure), history of psychiatric disorders, history of major trauma, and severe physical illnesses.

### 2.2 Sample size calculation

Kendall’s principle recommends a sample size of 5–10 times the number of study variables to meet the requirements for multivariate analysis (8). This study included 28 variables related to general demographic information, disease characteristics, self-efficacy, coping styles, family resilience, and social support. The sample size range was adjusted to 168–336 to account for a possible 20% rate of invalid questionnaire responses.

The sample size for the study was determined using Green’s formula for multiple linear regression. The formula used was *N* ≥ 50 + 8 m, where m is the number of predictor variables (9). The sample size was determined based on *α* = 0.05, (1-*β*) = 0.2, and the moderate effect sizes of the predictive factors for PTSD. The sample size was 242. The final sample size was 291 after accounting for a 20% invalid response rate.

The study distributed 340 questionnaires and received 312 valid responses, corresponding to a valid response rate of 91.8%. The effective sample size met and exceeded the minimum requirements estimated using both methods, indicating its adequacy for subsequent statistical analysis.

### 2.3 Research tools

The General Information Questionnaire was administered. It included demographic and disease-related information.

The Poison Severity Score (PSS) was used to assess poisoning severity (10, 11).

The General Self-Efficacy Scale (GSES) comprising 10 items was also used. Cronbach’s *α* was 0.916, indicating high internal consistency (12).

The Simple Coping Style Questionnaire (SCSQ) was used to assess positive and negative coping. Cronbach’s α was 0.846, indicating good internal consistency (13).

The Shortened Chinese Version of the Family Resilience Assessment Scale (FRAS-C) consisted of 32 items covering family communication and problem-solving, utilization of social resources, and maintenance of a positive outlook. Cronbach’s *α* was 0.916, indicating high internal consistency (14).

The Perceived Social Support Scale (PSSS) consisted of 12 items under three subscales: family, friends, and significant other. Cronbach’s *α* was 0.925, indicating high internal consistency (15).

The PTSD Checklist (PCL) consisted of 20 items. The cutoff score of 33 was used to distinguish between the PTSD symptom and non-symptom groups. Cronbach’s α was 0.958, indicating high internal consistency (16).

### 2.4 Data collection and ethics

The first data collection was conducted in the observation room of the Department of Poisoning and Occupational Diseases in the emergency department. The general information questionnaires for patients with acute poisoning were completed, and their PSS scores were determined. The second data collection was conducted during outpatient follow-up examinations 3 months after poisoning, and the remaining questionnaires were completed. The hospital ethics committee approved this study, and all participants provided informed consent.

### 2.5 Statistical analysis

Statistical analyses were performed using SPSS 26.0. Quantitative data are expressed as mean ± standard deviation, and categorical data are expressed as frequency and percentage. T-tests, one-way analysis of variance, Spearman correlation analysis, and multiple linear regression analysis were performed. Statistical significance was set at *p* < 0.05.

## 3 Results

### 3.1 General characteristics of the study participants

Of the 340 questionnaires distributed, valid responses were received for 312, corresponding to a valid response rate of 91.8%. Of the 312 patients with unintentional acute poisoning, 144 (46.2%) were male and 168 (53.8%) were female. Most (151, 48.4%) were aged 18–34 years. The highest proportions of cases were for junior high school (106, 34.0%) and senior high school (109, 34.9%) education. Farmers accounted for the largest number of cases (116, 37.2%). Most households had a monthly income of 4,001–7,000 yuan (162, 51.9%). Self-payment was the primary method of medical payment (132, 42.3%). The top three types of toxic substances were harmful gases (71, 22.8%), pesticides (61, 19.6%), and alcohol (52, 16.7%). Moderate and severe poisoning were observed in 173 (55.4%) and 70 (22.4%) patients, respectively. Thirty-four (10.9%) had a history of chronic disease. Mechanical ventilation and blood purification therapy were administered to 53 (17.0%) and 82 (26.3%) patients, respectively.

### 3.2 Current status of PTSD in patients with unintentional acute poisoning

**Figure 1:**
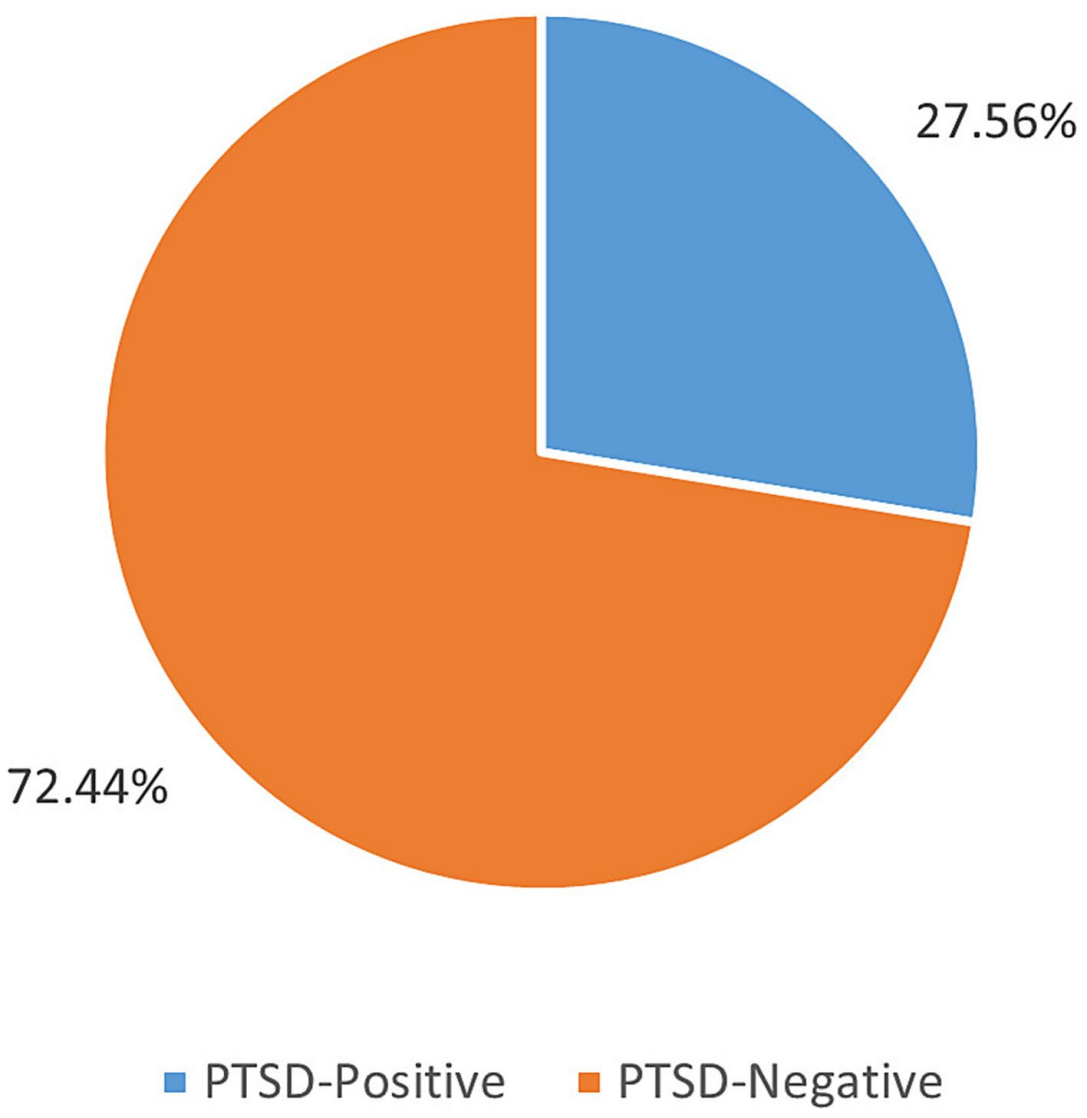
The positive rate of PTSD in patients with unintentional acute poisoning.

86 of the 312 patients in this study had PTSD (PCL-5 total score ≥ 33), corresponding to a positivity rate of 27.56% (Figure 1). The mean PCL-5 total score for the patients was 26.25 ± 16.49. The subscale scores, ranked from highest to lowest, were as follows: Avoidance Symptoms (1.37 ± 0.95), Negative Alterations in Cognition and Mood (1.32 ± 0.89), Alterations in Arousal and Reactivity (1.32 ± 0.89), and Intrusion Symptoms (1.26 ± 0.80).

### 3.3 Univariate analysis of PTSD in patients with unintentional acute poisoning

Univariate analysis revealed statistically significant differences in the total PTSD scores of the patients based on their educational levels, monthly household incomes, occupations, methods of medical payment, types of toxic agents, histories of chronic disease, mechanical ventilation, blood purification treatment, and poisoning severity (all *p* < 0.05) (Table 1). The total PTSD scores were higher for the patients with lower educational attainment, lower monthly household income, out-of-pocket medical payment, pesticide exposure, history of chronic disease, mechanical ventilation or blood purification therapy, and more severe poisoning. Gender, age, marital status, ethnicity, and place of residence had no statistically significant effect on the total PTSD scores (all *p* > 0.05).

**Table 1:** Univariate between-group comparisons of PTSD symptom scores according to demographic and clinical characteristics (*n* = 312).

Variables	*n*	Intrusion symptoms	Avoidance symptoms	Negative alterations in cognition/mood	Hyperarousal symptoms	PTSD total score
Gender						
Male	144	6.36 ± 3.58	2.73 ± 1.87	9.24 ± 5.74	7.87 ± 4.58	26.19 ± 14.84
Female	168	6.29 ± 4.34	2.75 ± 1.91	9.28 ± 6.58	7.99 ± 5.96	26.30 ± 17.83
t/P		0.166/0.868	0.097/0.923	0.062/0.951	0.197/0.844	0.058/0.954
Age						
18–34 years	151	6.28 ± 3.83	2.82 ± 1.82	9.15 ± 5.77	7.84 ± 5.18	26.10 ± 15.65
35–44 years	47	6.55 ± 4.17	2.66 ± 2.01	8.91 ± 5.72	7.47 ± 4.64	25.60 ± 15.39
45–59 years	76	6.59 ± 4.30	2.82 ± 2.01	10.24 ± 7.49	8.79 ± 6.45	28.43 ± 19.33
≥60 years	38	5.63 ± 3.88	2.37 ± 1.79	8.16 ± 5.48	7.16 ± 4.37	23.32 ± 14.85
F/P		0.547/0.650	0.650/0.584	1.096/0.351	1.044/0.373	0.873/0.455
Marital status						
Unmarried	97	6.57 ± 3.74	2.85 ± 1.84	9.68 ± 5.39	8.32 ± 5.22	27.41 ± 15.07
Married	195	6.05 ± 4.13	2.63 ± 1.93	8.86 ± 6.53	7.54 ± 5.45	25.07 ± 17.14
Divorced	11	8.09 ± 3.67	3.82 ± 1.40	11.55 ± 6.20	10.27 ± 4.69	33.73 ± 14.42
Widowed	9	7.33 ± 4.36	2.78 ± 1.79	10.67 ± 6.82	9.44 ± 5.34	30.33 ± 17.85
F/P		1.332/0.264	1.542/0.204	1.079/0.358	1.463/0.225	1.426/0.235
Ethnicity						
Han	307	6.29 ± 4.01	2.74 ± 1.89	9.23 ± 6.18	7.90 ± 5.37	26.17 ± 16.50
Ethnic minority	5	8.00 ± 3.24	3.00 ± 1.87	10.80 ± 7.46	9.80 ± 5.26	31.60 ± 16.59
t/P		0.946/0.345	0.309/0.757	0.560/0.576	0.785/0.433	0.730/0.466
Residence						
Rural	197	6.60 ± 4.01	2.81 ± 1.92	9.53 ± 6.20	8.25 ± 5.51	27.20 ± 16.55
Town	68	6.25 ± 3.61	2.87 ± 1.70	9.57 ± 5.79	8.19 ± 4.51	26.88 ± 14.67
Urban	47	5.23 ± 4.37	2.26 ± 1.99	7.68 ± 6.62	6.21 ± 5.64	21.38 ± 18.14
F/P		2.257/0.106	1.856/0.158	1.808/0.166	2.885/0.057	2.445/0.088
Education level						
Primary school or below	43	8.23 ± 4.89	3.33 ± 2.11	11.91 ± 8.47	10.28 ± 7.45	33.74 ± 21.75
Junior high school	106	6.72 ± 4.15	2.89 ± 1.96	9.54 ± 6.27	8.26 ± 5.38	27.41 ± 16.84
Senior high school	109	5.61 ± 3.32	2.51 ± 1.67	8.25 ± 5.08	7.06 ± 4.36	23.43 ± 13.43
College or above	54	5.46 ± 3.64	2.44 ± 1.90	8.65 ± 5.45	7.17 ± 4.67	23.72 ± 14.84
F/P		5.874/0.001	2.591/0.053	3.942/0.009	4.335/0.005	4.788/0.003
Occupation						
Unemployed	3	5.33 ± 0.58	2.00 ± 1.00	8.67 ± 3.51	6.67 ± 0.58	22.67 ± 5.13
Farmer	116	6.92 ± 4.54	2.89 ± 2.19	10.18 ± 7.17	8.79 ± 6.17	28.78 ± 19.02
Worker	54	5.52 ± 3.63	2.48 ± 1.41	7.98 ± 4.74	6.70 ± 3.93	22.69 ± 12.66
Self-employed	46	6.50 ± 3.89	3.09 ± 1.86	10.04 ± 6.31	8.76 ± 5.36	28.39 ± 16.64
Corporate staff	29	6.17 ± 4.11	2.97 ± 2.11	8.90 ± 6.90	8.31 ± 6.25	26.34 ± 18.72
Student	55	6.36 ± 3.19	2.58 ± 1.46	8.95 ± 4.57	7.22 ± 3.88	25.11 ± 12.00
Other	9	3.00 ± 1.87	1.11 ± 1.27	4.33 ± 3.81	3.56 ± 4.07	12.00 ± 9.99
F/P		1.920/0.077	1.903/0.080	1.963/0.071	2.433/0.026	2.247/0.039
Monthly household income (CNY)						
≤4,000	80	7.13 ± 4.45	2.93 ± 2.00	10.26 ± 6.98	8.70 ± 6.40	29.01 ± 18.59
4,001–7,000	162	6.53 ± 3.84	2.97 ± 1.91	9.71 ± 6.10	8.48 ± 5.08	27.69 ± 16.04
7,001–10,000	27	5.41 ± 4.02	2.33 ± 1.62	8.07 ± 6.64	6.56 ± 5.18	22.37 ± 16.70
>10,000	43	4.60 ± 3.10	1.79 ± 1.44	6.44 ± 3.33	5.30 ± 3.12	18.14 ± 10.22
F/P		4.480/0.004	5.297/0.001	4.420/0.005	5.372/0.001	5.342/0.001
Medical payment method						
Self-paid	132	7.24 ± 4.12	3.11 ± 2.03	10.73 ± 6.89	9.23 ± 5.92	30.33 ± 17.97
New rural cooperative	45	5.84 ± 3.98	2.56 ± 2.08	8.60 ± 5.77	7.49 ± 5.12	24.49 ± 16.13
Employee medical insurance	33	4.15 ± 3.46	1.94 ± 1.89	6.52 ± 5.98	5.85 ± 5.77	18.45 ± 16.45
Resident medical insurance	30	5.63 ± 3.93	2.43 ± 1.76	8.87 ± 6.24	7.10 ± 5.03	24.03 ± 16.31
Commercial insurance	33	7.33 ± 3.17	3.12 ± 1.54	7.76 ± 4.88	8.46 ± 4.32	28.67 ± 12.86
Work-related injury insurance	39	5.26 ± 3.63	2.28 ± 1.10	7.23 ± 3.57	6.00 ± 2.53	20.77 ± 9.30
F/P		4.914/<0.001	3.307/<0.01	3.980/<0.01	4.023/<0.01	4.548/<0.01
Chronic disease history						
No	278	6.13 ± 3.82	2.67 ± 1.85	8.85 ± 5.68	7.65 ± 4.87	25.30 ± 15.24
Yes	34	7.88 ± 5.08	3.29 ± 2.14	12.65 ± 8.84	10.24 ± 8.11	34.06 ± 23.29
t/P		1.946/0.059	1.817/0.070	2.477/0.019	1.818/0.077	2.138/0.039
Type of poison						
Pesticide	61	9.20 ± 4.60	3.89 ± 2.17	13.93 ± 7.85	12.15 ± 6.71	39.16 ± 20.14
Drug poisoning	41	5.15 ± 3.30	2.44 ± 1.79	7.34 ± 5.25	6.49 ± 4.33	21.41 ± 13.42
Industrial chemicals	35	5.20 ± 3.56	2.17 ± 1.15	6.91 ± 3.15	5.74 ± 2.38	20.03 ± 8.70
Poisonous plants/animals	36	4.69 ± 3.23	2.08 ± 1.48	7.14 ± 4.56	6.06 ± 3.81	19.97 ± 12.29
Harmful gases	71	6.20 ± 3.81	2.77 ± 2.02	9.48 ± 5.88	8.00 ± 4.96	26.45 ± 15.82
Alcohol	52	6.15 ± 2.62	2.52 ± 1.52	8.12 ± 3.79	6.83 ± 3.88	23.62 ± 10.71
Other	16	5.56 ± 5.61	2.44 ± 2.00	9.00 ± 8.18	7.87 ± 7.15	24.88 ± 22.35
F/P		8.488/<0.001	5.855/<0.001	9.690/<0.001	10.476/<0.001	10.345/<0.001
Mechanical ventilation						
No	259	5.74 ± 3.58	2.48 ± 1.76	8.47 ± 5.34	7.20 ± 4.61	23.90 ± 14.29
Yes	53	9.17 ± 4.69	4.00 ± 2.01	13.09 ± 8.37	11.51 ± 7.11	37.77 ± 21.21
t/P		5.036/<0.001	5.580/<0.001	3.859/<0.001	4.231/<0.001	4.556/<0.001
Blood purification						
No	230	5.73 ± 3.65	2.54 ± 1.73	8.35 ± 5.31	7.16 ± 4.62	23.78 ± 14.33
Yes	82	7.99 ± 4.46	3.29 ± 2.20	11.82 ± 7.65	10.10 ± 6.61	33.20 ± 19.94
t/P		4.127/<0.001	2.795/<0.001	3.793/<0.001	3.715/<0.001	3.930/<0.001
Poisoning severity						
Mild	69	5.51 ± 2.36	2.35 ± 1.15	7.87 ± 3.07	6.67 ± 2.23	22.39 ± 7.30
Moderate	173	5.73 ± 3.69	2.50 ± 1.78	8.47 ± 5.52	7.13 ± 4.85	23.84 ± 14.81
Severe	70	8.57 ± 5.11	3.71 ± 2.37	12.59 ± 8.54	11.16 ± 7.23	36.03 ± 26.35
F/P		15.730/<0.001	13.104/<0.001	14.376/<0.001	18.345/<0.001	17.781/<0.001

Data are presented as mean ± standard deviation. For comparisons between two groups, independent-samples *t*-test was used; for comparisons among three or more groups, one-way ANOVA was used. *t* or F values and corresponding *p* values are shown.

### 3.4 Scores of psychosocial variables and their correlation with PTSD

The patients had a mean general self-efficacy score of 23.80 ± 6.73, indicating a moderate level. Their total social support score was 59.77 ± 12.49, reflecting moderate support. The highest scores were for the family support dimension. Their mean total family resilience score was 94.64 ± 13.49. Their positive and negative coping style scores were 19.00 (14.00, 25.75) and 10.00 (7.00, 13.00), respectively. Spearman correlation analysis revealed significant negative correlations between the PTSD total scores and general self-efficacy (*r* = −0.996, *p* < 0.01), total social support (*r* = −0.342, *p* < 0.01), total family resilience score (*r* = −0.327, *p* < 0.01), and positive coping (*r* = −0.320, *p* < 0.01). It also revealed a significant positive correlation with negative coping (*r* = 0.155, *p* < 0.01).

### 3.5 Multiple linear regression analysis of factors influencing PTSD in patients with unintentional acute poisoning

Stepwise multiple linear regression analysis was performed using the variables that were statistically significant in the univariate and correlation analyses as the independent variables and the total PTSD score as the dependent variable. The five variables included in the regression equation were medical payment method (employment injury insurance), chronic disease history, poisoning severity, general self-efficacy, and social support. The model was statistically significant (*F* = 378.124, *p* < 0.001) and had an adjusted *R^2^* of 0.885, indicating that these variables collectively explained 88.5% of the variance in the total PTSD scores. General self-efficacy (*β* = −0.909), social support (*β* = −0.059), and employment injury insurance (*β* = −0.043) were protective factors for PTSD, whereas chronic disease history (*β* = 0.060) and poisoning severity (*β* = 0.101) were risk factors. The detailed results are provided in Table 2.

**Table 2:** Results of multiple linear regression analysis of factors influencing PTSD in patients with unintentional acute poisoning (*n* = 312).

Item	*B*	*SE*	*β*	*t*	*P*
(Constant)	80.595	2.091		38.544	< 0.001
General self-efficacy	−2.227	0.052	−0.909	42.587	< 0.001
Poisoning severity	2.318	0.447	0.101	5.184	< 0.001
Chronic medical history	3.174	1.032	0.06	3.075	0.002
Social support	−0.078	0.028	−0.059	−2.812	0.005
Employment injury insurance	−2.466	1.21	−0.043	−2.038	0.042

Adjusted *R^2^* = 0.881, *R^2^* = 0.885, *p* <0.001. To include categorical variables in the multiple linear regression model, the following coding was applied: chronic medical history (no = 0, yes = 1); poisoning severity (mild = 1, moderate = 2, severe = 3); for medical payment method, dummy variables were created with “self-paid” as the reference category, among which employment injury insurance was coded as 1 (yes) or 0 (no). General self-efficacy and social support were continuous variables and retained their original scores.

## 4 Discussion

This study explored the current status of PTSD among patients with unintentional acute poisoning and its influencing factors. The results indicated a positive detection rate of 27.56% for PTSD in these patients, which was significantly higher than that in the general population. This highlights acute poisoning events as an important source of psychological trauma (17). Poisoning events are often sudden and life-threatening, and their treatment involves invasive procedures (such as mechanical ventilation) and highly uncertain outcomes. These factors collectively constitute a potent stressor and align with the pathophysiological basis for PTSD development (18).

This study found that social support and general self-efficacy are protective factors against PTSD. This is consistent with the findings of several studies involving other trauma populations. Social support, especially emotional and substantive support from family and friends, can mitigate the impact of stressful events to some extent (19). It helps individuals reduce feelings of isolation and helplessness, lower their assessment of event threat by enhancing their perceived ability to cope with trauma, reduce negative evaluations of the trauma, and alleviate harmful physiological responses. These effects reduce intrusive memories and avoidance symptoms (20). General self-efficacy reflects the confidence of an individual in coping with challenges and maintaining control. Patients with high self-efficacy are more likely to view recovery as a manageable challenge rather than an insurmountable threat. This leads to the more proactive adoption of positive coping strategies, adherence to treatment, and greater psychological resilience for self-regulation when facing avoidance or anxiety (21). These two factors complement each other and jointly build a psychological defense against PTSD.

The study further highlights the objective severity of poisoning as an independent risk factor for PTSD. Severe poisoning is associated with a greater threat to the patient’s life, more marked physiological impact, and more complex and prolonged subsequent treatment. These factors contribute to intense traumatic memories. Chronic medical history is another risk factor that indicates the cumulative effect of pre-existing disease burden. Patients with chronic diseases have weaker physiological reserves, more pronounced treatment conflicts, and greater anxiety about the consequences of their illness. These factors can exacerbate their mental stress and psychological trauma and increase their susceptibility to PTSD following unintentional acute poisoning of comparable severity (22).

Employment injury insurance has a protective effect. This is primarily attributed to the significant alleviation of the financial pressure and uncertainty faced by patients and their families when they are confronted with sudden severe illness (23). The relief of the financial burden directly reduces medical cost anxiety, a major secondary stressor associated with illness. It enables patients to focus more on physical recovery rather than be consumed by persistent fears of “falling into poverty due to illness.” This payment method also facilitates timely, effective, and high-quality treatment services for patients with acute poisoning. Employment injury insurance objectively creates a more conducive environment for psychological recovery.

This study innovatively proposes to focus on PTSD in a specific population with unintentional acute poisoning. It broadens the scope of PTSD research and enriches the literature on PTSD in this population. The findings have clear implications for clinical practice. Medical workers should routinely assess the psychological states of patients during emergency care and subsequent follow-ups. They should pay attention to those with severe poisoning, comorbid chronic diseases, poor perceived social support, and low self-efficacy. Efforts should be made to enhance the self-efficacy of patients with unintentional acute poisoning through psychological support and sharing of success stories to facilitate their transition from passive victims to active participants in their recovery. However, systematically building social support networks by encouraging family involvement, fostering peer support, and integrating professional psychological therapy is crucial to creating an inclusive and supportive rehabilitation environment for patients.

This study has several limitations. First, its cross-sectional design precludes the establishment of causal relationships, and complex bidirectional influences may exist between PTSD and variables such as self-efficacy and social support. Second, the sample was drawn from a single tertiary hospital, which limits the representativeness of the findings for all patients with unintentional acute poisoning in the country and restricts their generalizability. Third, some subgroups (e.g., ethnic minorities, divorced/widowed, unemployed) had small sample sizes, which may limit the precision of estimates for those groups. In addition, the following methodological limitations should be acknowledged. (a) We did not report the number of patients with PTSD (PCL-5 ≥ 33) within each subgroup, which limits the clinical interpretability of subgroup differences. (b) Although all measurement tools have demonstrated validity in previous studies, we did not independently conduct validity tests (e.g., exploratory factor analysis) in this specific population. Based on the limitations above, future research should adopt longitudinal, multicenter designs with adequate subgroup sample sizes, report PTSD rates by subgroup using a validated cut-off of PCL-5 ≥ 33, and consider validity testing of measurement tools in this specific population.

## 5 Conclusion

Patients with unintentional acute poisoning, especially those with severe poisoning or underlying chronic conditions, have a higher incidence of PTSD. Clinicians should implement early PTSD screening and provide psychological interventions focused on enhancing social support and self-efficacy. Psychological counseling should be integrated into treatment, and family and social support systems should be strengthened. Medical cost coverage should also be optimized to promote the physical and mental recovery of these patients.

## Data Availability

The raw data supporting the conclusions of this article will be made available by the authors, without undue reservation.

## References

[1] Li L Shi C Gao Y Yang Y. Analysis of adverse events and psychological status in 71 patients with acute organophosphorus pesticide poisoning. J Clin Med Pract. (2022) 26:35–44. doi: 10.7619/jcmp.20221700

[2] National Health and Family Planning Commission of the People’s Republic of China. GBZ/T 300.1–2017 Determination of Toxic Substances in Workplace air—Part 1: General Principles. Beijing: People’s Medical Publishing House (2018).

[3] Editorial Committee of China Health Statistics Yearbook. China Health Statistics Yearbook. Beijing: Peking Union Medical College Press (2018).

[4] American Psychiatric Association. "Posttraumatic stress disorder". In: Diagnostic and Statistical Manual of Mental Disorders. Washington: American Psychiatric Publishing (2013)

[5] Ontario Health (Quality). Internet delivered cognitive behavioural therapy for post traumatic stress disorder or acute stress disorder: a health technology assessment. Ont Health Technol Assess Ser. (2021) 21:1–120. PMC8398719 34527087

[6] Wang X Yu W. Nursing intervention for a patient with post traumatic stress disorder after liquid ammonia poisoning. Chin Folk Ther. (2015) 23:84–5.

[7] Li M Hu H Hu J Wang T Li Y Zhang L . Psychological analysis of post traumatic stress disorder in hospitalized patients after the Chongqing Kaixian natural gas blowout accident. Chin J Clin Rehabil. (2004) 27:5772–3.

[8] Fang J. Modern Medical Statistics. Beijing: People’s Medical Publishing House (2002).

[9] Green SB. How many subjects does it take to do a regression analysis? Multivariate Behav Res. (1991) 26:499–510. doi: 10.1207/s15327906mbr2603_7, 26776715

[10] Chinese College of Emergency Physicians, Committee of Poisoning and Treatment of Chinese Society of Toxicology. Chinese expert consensus on diagnosis and treatment of acute poisoning. Chin J Emerg Med. (2016) 25:1361–75. doi: 10.3760/cma.j.issn.1671-0282.2016.11.004

[11] Wang J. Value of poisoning severity score in condition assessment and prognosis judgment of patients with acute poisoning. China Pract Med. (2020) 15:31–3.

[12] Schwarzer R Bassler J Kwiatek P Schröder K Zhang JX. The assessment of optimistic self beliefs: comparison of the German, Spanish, and Chinese versions of the general self efficacy scale. Appl Psychol. (1997) 46:69–88. doi: 10.1080/026999497378557

[13] Xie Y. A preliminary study on the reliability and validity of the simplified coping style questionnaire. Chin J Clin Psychol. (1998) 6:53–4.

[14] Li Y Zhao Y Zhang J Lou F Cao F. Psychometric properties of the shortened Chinese version of the family resilience assessment scale. J Child Fam Stud. (2016) 25:2710–7. doi: 10.1007/s10826-016-0432-7

[15] Wang F Huang L Zhang H Jiang H Chang X Chu Y. The mediating role of perceived stress on the relationship between perceived social support and self care ability among Chinese enterostomy patients. Support Care Cancer. (2021) 29:3155–62. doi: 10.1007/s00520-020-05829-8, 33074359

[16] Bovin MJ Marx BP Weathers FW Gallagher MW Rodriguez P Schnurr PP . Psychometric properties of the PTSD checklist for diagnostic and statistical manual of mental disorders–fifth edition (PCL-5) in veterans. Psychol Assess. (2016) 28:1379–91. doi: 10.1037/pas0000254, 26653052

[17] Berger W Coutinho ES Figueira I Marques-Portella C Luz MP Neylan TC . Rescuers at risk: a systematic review and meta regression analysis of the worldwide current prevalence and correlates of PTSD in rescue workers. Soc Psychiatry Psychiatr Epidemiol. (2012) 47:1001–11. doi: 10.1007/s00127-011-0408-2, 21681455 PMC3974968

[18] Wang Y Wu X Lei G. Investigation and analysis of post traumatic stress disorder syndrome in emergency critically ill patients. Chin Hosp Stat. (2018) 25:384–7.

[19] Ozer EJ Best SR Lipsey TL Weiss DS. Predictors of posttraumatic stress disorder and symptoms in adults: a meta analysis. Psychol Bull. (2003) 129:52–73. doi: 10.1037/0033-2909.129.1.52, 12555794

[20] Zalta AK Tirone V Orlowska D Blais RK Lofgreen A Klassen B . Examining moderators of the relationship between social support and self reported PTSD symptoms: a meta analysis. Psychol Bull. (2021) 147:33–54. doi: 10.1037/bul0000316, 33271023 PMC8101258

[21] Bidzan M Bidzan-Bluma I Szulman-Wardal A Stueck M Bidzan M. Does self-efficacy and emotional control protect hospital staff from COVID-19 anxiety and PTSD symptoms? Psychological functioning of hospital staff after the announcement of COVID-19 coronavirus pandemic. Front Psychol. (2020) 11:552583. doi: 10.3389/fpsyg.2020.552583, 33424673 PMC7785971

[22] Wang Z Zhao W. Review and prospect of chronic disease prevention and control in China. Chin J Dis Control Prev. (2019) 23:1025–8.

[23] Zhang Y. Study on the Indicators Influencing the Discharge Willingness of Medical Insurance Trauma Patients. Changsha: Central South University (2013).

